# Supplementary material for: Irreversible Electroporation Mediates Glioma Apoptosis via Upregulation of AP-1 and Bim: Transcriptome Evidence
Source: Brain Sci. 2022 Oct 29;12(11):1465. doi: 10.3390/brainsci12111465 (PMC9688309; doi:10.3390/brainsci12111465)
Supplement: Supplementary file 1 [file brainsci-12-01465-s001.zip › brainsci-1954767-supplementary.pdf]

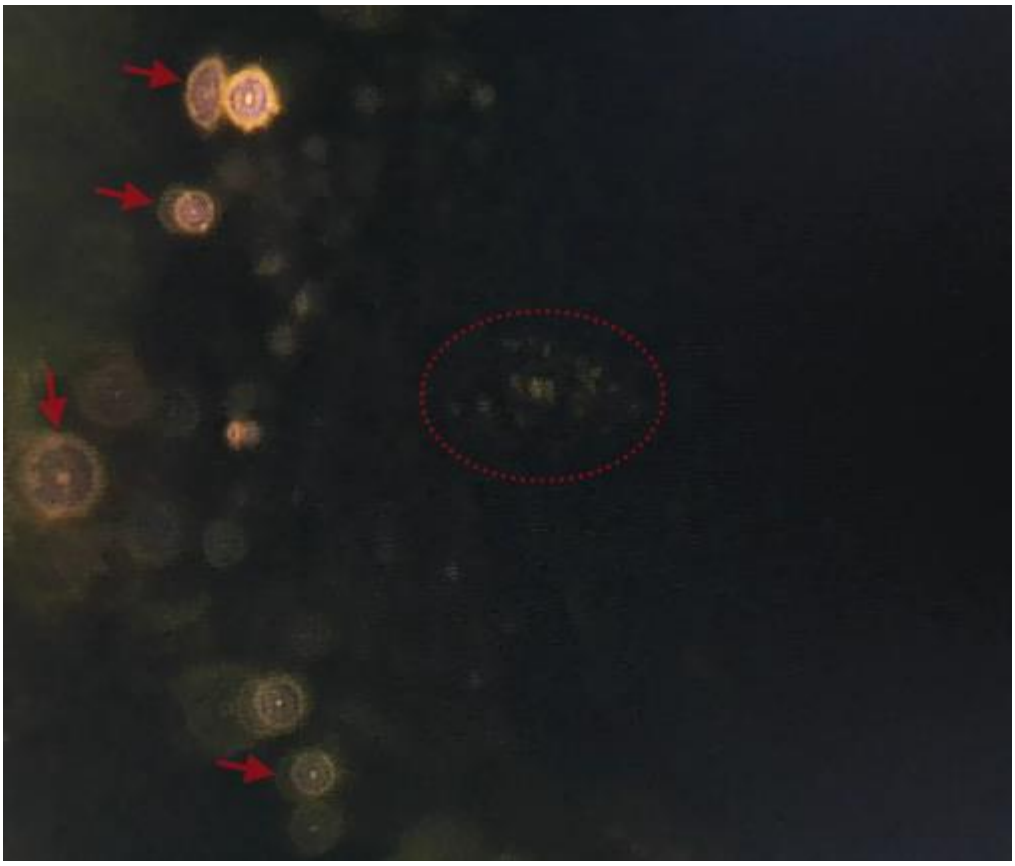

Figure S1. Light microscopy picture of gas bubbles formation around the cathodal needle electrode. Red dotted ellipse indicates the electrode (Diameter: 0.4mm). Red arrows indicate gas bubbles.
